# Atmospheric Germs and Their Relation to Disease

**Published:** 1871-04

**Authors:** Frank H. Davis


CHICAGO
MEDICAL EXAMINER:

N. S. DAVIS, M.D., EDITOR.
F. H. DAVIS, ASSISTANT-EDITOR.

VOL. XII. APRIL, 1871. NO. 4.

### Original Contributions.

### ATMOSPHERIC GERMS AND THEIR RELATION TO DISEASE.

By FRANK H. DAVIS, M.D.

Notwithstanding the time and attention which the ablest scientific observers have devoted to investigation of the subject, we have still very little definite satisfactory knowledge of the nature and character of the causes originating many of our most common and best understood diseases. It is not probable that the possession of this knowledge would be of any practical assistance in the treatment of disease, but as a key to the proper hygienic measures to be adopted, an understanding of the true source and nature of these causes, or influences, would be of the very greatest importance. By the neutralization or destruction of the poisonous material, or at least the avoidance of exposure to their influence, we would, in many instances, be enabled to avoid the contraction of disease, and could effectually prevent the extension of the contagious or epidemic diseases. The discovery of the efficiency of vaccination, and the method of isolation, now practiced with all contagious diseases, has reduced unmeasurably the ravages of that class of affections. The epidemic diseases, however, of which cholera is a prominent example, are much less easily controlled. Isolation is of no avail. Exposure to foul air and unhealthy surroundings, where the poisonous effluvia, from more or less decomposing material, is being constantly inhaled, being probably the method in which these diseases are contracted, and not to any exposure to the persons diseased. When the discharges from cholera patients are allowed to remain and decompose in the room, or are thrown in an open place, where they contaminate the atmosphere, they will, of course, exert the same poisonous influence as any other putrescent material.

In searching for the origin of the various diseases, wherever no other reasonable tangible cause could be assigned, atmospheric impurities or poisons have usually been held responsible. It is a well understood fact that persons affected with certain contagious diseases, as variola, rubeola, and scarlatina, contaminate the atmosphere for a certain distance around them, with a specific poison capable of propagating the particular disease by which it is engendered. These poisons, however, are of so subtle a nature as to have eluded every attempt at actual demonstration, and we can only prove their existence by their effects. An examination, even, of the poisonous material of the variola pustle, which is known to be capable of engendering the disease, affords us no satisfactory explanation of its peculiar properties. Prof. Lionel S. Beale, in a recent article in the *London Microscopical Journal*, says: "I have examined the contents of the little vesicle which rises in small-pox at different stages of its development, and find, as in allied pathological changes, vast multitudes of minute particles of living matter or bioplasm, but, as will have been anticipated from what has been already said, these present nothing peculiar or characteristic, nothing that would enable us to say, if we saw these particles under the microscope, that they were obtained from a small-pox vesicle, and would certainly give rise to that disease."

The germ theory attempts to account for the origin of these diseases by supposing the existence of certain specific germs, of either a vegetable or animal nature, which, floating in the atmosphere, are introduced into the system with the inspired air. As the most rational and probable explanation that has been offered to account for the occurrence of these phenomena, this theory has been very generally accepted by the scientific world. Microscopists have failed, however, as yet, to demonstrate the existence of any such specific germs holding a definite relation to the origin of disease. Organic germs are found to be present, in more or less abundance, in all atmospheres. The large amount of solid material present in air apparently perfectly clear and pure, is most beautifully shown by the very common and familiar phenomena which occurs when rays of sunlight enter a darkened or partially-darkened room, through a narrow opening in a blind or curtain. A cloud of very minute dust-particles, floating about and glistening in the sunlight, mark the passage of the rays through the atmosphere, the particles being perfectly invisible anywhere outside of their course. Prof. Tyndell has proved these invisible dust-clouds to be made up mostly of organic material, by the very simple experiment of introducing the flame of a spirit-lamp into the rays, when most of the particles were consumed and a dark smoke produced. This, however, by no means proves the particles to be *living* germs, and being apparently normally, or at any rate constantly, present in the atmosphere, *they* cannot, of course, be considered as holding any causative relation to disease.

The occurrence of suppurations, of erysipelatous inflammations, gangrene, etc., in wounds after surgical operations, and in abscesses which have been opened and their cavities exposed to the air, is also attributed by many surgeons to the absorption of living germs from the air. In accordance with this theory, an antiseptic mode of treatment has been devised by Prof. Lister, and since modified and improved upon by Prof. E. Andrews, of this city. The treatment consists, essentially, in the exclusion of the suppurating surfaces from contact with the air, wherever possible, and in the thorough application of carbolized solutions and dressings to such wounds and abscesses as have been exposed to the atmosphere, carbolic acid being supposed to possess the power of destroying all organic germs.

This antiseptic treatment has been thoroughly tested by Prof. Andrews in the wards of Mercy Hospital, and its efficacy fully established by the results obtained. Do these favorable results, however, necessarily confirm the correctness of the germ theory? Or, are not the injurious effects resulting from the admission of air to wounds and abscesses quite as readily accounted for by other equally probable theories? May not, for instance, the organic matter thrown off from the lungs and the cutaneous surface, when retained in close and badly-ventilated rooms, or in crowded hospital wards, until it has undergone decomposition, be of itself capable of poisoning not only the exposed wounds, but also the blood and system generally of the patients compelled to breathe the atmosphere thus contaminated? In support of this theory we have the testimony of some experienced surgeons, Dr. Skey, of St. Bartholomew's, for example, who still claims that the presence of air in wounds is entirely inocuous where the patient's room is properly ventilated.

The results of numerous careful microscopical examinations of the ordinary atmosphere, and also of various special atmospheres, have been published in the *London Microscopical Journal* during the past year. These examinations, although productive only of negative results, as regards the disease-germ theory, have, nevertheless, led to the discovery of some curious and interesting facts in regard to atmospheric impurities.

In the atmosphere of a dissecting hall, according to the observations of George Seigerson, M.D., "Fragments of fibres were found present, with the marks of the scalpel on them! There were fibrils of voluntary and of involuntary muscles of white and yellow fibrous tissues; some epithelial scales, fat cells, corpuscles, fine fragments of hair, and inchoate particles. It was a somewhat ghostly revelation; but might be of advantage by inducing those in authority to ventilate thoroughly and disinfect daily." The same observer also says, "Tobacco-smoke, with some difficulty, was got under the microscope. It was examined on entering and on leaving the mouth. Little globules of nicotine were discovered twirling and flitting about in it, like monods. Some remained on the walls of the mouth; when the smoke is breathed (by novices) more globules are retained in the lungs, and nausea and illness supervene. These globules, if found in the air, distributed by cigar-smokers, might be taken for germs, as they would resist the iodine-test for amyloids."

In the atmosphere of an iron-foundry, iron was found to be present in the form of balls. "These were found to be hollow, and the fragments of their shells were discovered to be translucent. These iron bombs, or balloons, varied from $\frac{1}{500}$th of an inch to $\frac{1}{5000}$th having been measured with the micrometer. The diameter of a bomb of $\frac{1}{2000}$th of an inch was computed to be sixteen times greater than the thickness of the shell, which would therefore be about the $\frac{1}{30000}$th of an inch. The computation was made in order to find at what degree of thinness its iron material was translucent. There were no spores or seeds present, no fibres at all, except a few cotton filaments from the garments of artisans, fibrous carbon particles, and a specimen of contorted, branchy metal. There were no germs here, yet the sunbeams were full of dancing motes. The ray shining on them, assumed, in consequence of their hue, a bluish color, similar to that observed when the carboniferous smoke of a candle or lamp is placed in the track of a sunbeam. On entering such an atmosphere, the taste of carbon, and, indeed, of iron, may be readily perceived."

During the past few months, I have, myself, made a number of microscopic examinations of the atmosphere of rooms, in which patients laboring under different diseases, contagious and otherwise, were lying, and have also examined carefully the breath of the patients themselves. These examinations were conducted in the following manner: A drop of clear, pure glycerine was placed upon a perfectly clean glass slide, which had been carefully excluded from contact with the air outside of the sick-room. The slide thus prepared was placed upon a shelf, a window-sill, or any convenient place, where it could remain undisturbed for twenty-four hours. The currents of air passing through the room brought more or less of the dust and floating particles in contact with the glycerine, by which they were retained. A slide similarly prepared was then held, for a minute or two, before the mouth and nostrils of the patient, so that the solid particles contained in the expired air might be caught upon it.

The observations were made in the presence of seven cases of typhoid and typho-malarial fevers, one case of erysipelas, one case of scarlatina, one of rubeola, and one case of diphtheria. The atmosphere of my own sleeping apartment, and of that of other rooms occupied by perfectly healthy persons, was submitted to a similar examination, as was also the breath of healthy persons.

The principal difficulty experienced in examining these slides was caused by the large amount and variety of the materials which the glycerine had entrapped. Particles of feathers from the beds or pillows, white cotton fibres from the sheets, red and blue woolen fibres, etc., were readily distinguised in most of the specimens. Numerous and various other particles, the nature of which we were unable to determine, but some of them easily capable of being construed into germs, were also present in every instance.

On a careful comparison of the slides exposed only to normal, healthy atmosphere, with those which had been exposed in the contaminated or contagious atmospheres, we were unable to detect the slightest particle of any kind in the one, which was not equally present in the other. The materials brought to view, therefore, whatever organic matter or germs may have been present amongst them, could not be considered as holding any relation to disease.

We regret that the pressure of other duties this winter has rendered it impossible for us to devote the time and study to this subject which we desired. The few hasty observations which have been made by us, are too incomplete to be of any interest or importance by themselves. Their negative results, however, are confirmatory of the conclusions arrived at by those who have given the subject most attention. We are forced therefore to conclude, either that the disease producing poisons, for which we have been searching, have eluded all the various contrivances devised for the purpose of bringing them under observation, or else, that these influences emanate from some of the normal excretory products, in which we may suppose certain changes to have taken place; changes which are, however, neither apparent to the eye, nor appreciable by any means yet discovered.

